# Effects of Severe Floods and Droughts on Wildlife of the Pantanal Wetland (Brazil)—A Review

**DOI:** 10.3390/ani2040591

**Published:** 2012-10-15

**Authors:** Cleber J. R. Alho, João S. V. Silva

**Affiliations:** 1 Graduate Program on Environment and Regional Development, Anhanguera-Uniderp University, Campo Grande, 79003-010, MS, Brazil; 2 Embrapa Informática Agropecuária, CNPTIA, Campinas, 13083-886, SP, Brazil; E-Mail: jvilla@cnptia.embrapa.br

**Keywords:** biodiversity, conservation, floodplain, Pantanal

## Abstract

**Simple Summary:**

The Pantanal is a wetland in the center of South America, (140,000 km² in Brazil), in the Upper Paraguay River Basin. Because of its diverse and abundant wildlife, it is recognized as one of the most important freshwater ecosystems in the world. Many endangered species occur there, including jaguar; waterfowl are exceptionally abundant. Relief varies between the low, and flat floodplain, and the surrounding non-flooded plateau areas. Rainfall shows inter-annual variability, influencing the flooding patterns. Historical climate instability of severe multi-annual flood and dry events has affected animals’ habitats as well as their community structure, population size and behavioral ecology.

**Abstract:**

Flooding throughout the Pantanal is seasonal. The complex vegetative cover and high seasonal productivity support a diverse and abundant fauna. A gradient in flood level supports a range of major habitats in a complex mosaic with annual seasonality. The rivers and streams are lined with gallery forests, and other arboreal habitats exist in the more elevated areas. The remainder is either grasslands or seasonally flooded grasslands. The regional flora and fauna are adapted to annual water fluctuation. However, an inter-annual series of higher or lower rainfalls has caused either severe floods or drastic dry seasons. Large scale climate phenomena such as greenhouse gases, El Niño and La Niña influence the seasonality of floods and droughts in the Pantanal. Knowledge of severe floods and droughts, which characterize natural disasters, is fundamental for wildlife management and nature conservation of the Pantanal. Plants and wild animals, for example, are affected by tree mortality in riparian forest after extreme flooding, with consequent habitat modification for wild animals. In addition, human activities are also affected since cattle ranching and ecotourism are economically important in the region, and when seasons with unusual floods or droughts occur, areas with human settlements are impacted.

## 1. Introduction

The Pantanal is a large tropical wetland (140,000 km², larger than the Everglades) located in the center of South America, in western Brazil’s Upper Paraguay River Basin (UPRB) (latitude 15°30'–22°30' south and longitude 54°45'–58°30' west). The UPRB is an area of 361,666 km^2^, within which the land ranging 200–1,200 meters above sea level is considered as highland or plateau *(Planalto)* and the flatland between 80 and 150 meters is the floodplain, called the Pantanal, which occupies an area of 138,183 km^2^, representing about 38% of the Basin [1,2]. Neighboring biomes biogeographically influence the Pantanal’s biodiversity; these are the savanna or *Cerrado* which covers the surrounding plateaus to the east, *Amazonia* to the north, the Atlantic Forest to the southeast, represented by semi-deciduous and deciduous forests, and the *Chaco* to the southwest. The Pantanal was declared a National Heritage Site by the Brazilian Constitution of 1988 and a Biosphere Reserve by UNESCO in 2000.

The Paraguay River originates in Brazilian territory, on the plateau Chapada dos Parecis in Mato Grosso State, and then heads southwards receiving important tributaries along its course. The following major rivers feeding the Pantanal are tributaries on the left bank of the Paraguay River, (from north to south): Bento Gomes, Cuiabá, São Lourenço-Itiquira, Taquari, Negro, Aquidauana-Miranda, Nabileque and Apa. The Paraguay River joins the Paraná River in southern Brazil and, united, they flow into the La Plata River. These rivers are slow moving when they meet the flatland of the Pantanal. Periodically, they overflow their banks. Combined, the fluctuating water levels, nutrients and wildlife form a dynamic ecosystem. Flooding may inundate some 80% of the entire Pantanal. The total area inundated at any given time fluctuated between 10,000 and 110,000 km^2^ between 1979–1987 [3]. In contrast, when the water returns to the riverbeds, during the dry season most of the flooded areas remain dry [1,2].

Annual average precipitation is 1,400 mm, varying between 800 and 1,600 mm, but in some years it can reach 2,000 mm. The rainfall is highly seasonal, occurring between October and March, but with variations between the northern and the southern regions [4].

The relationships between slope/soil/water/vegetation cover/fauna are significant and serve to differentiate the Pantanal flat plains from the surrounding plateaus. Annual rainfall in the highlands generally exceeds 1,200 mm, which produces a rapid response in the drainage basin. The reduced run-off capacity of the plains results in regional flooding. Depressions retain water, forming small temporary lakes and ponds (*baías*), or flooding the permanent ponds. During times of low water the retained water volume does not return to the river-bed to be drained, but remains where it is and either evaporates or infiltrates the soil [2]. During the high water level, the flow moves slowly through depressions locally known as “*corixos*” or along shallow water paths, “*vazantes*”.

Evaporation of water from the land is greater than the volume of water received through precipitation. The Pantanal is considered an enormous evaporation "window" [2]. The retention of water on the plains reaches 30 to 60%, transforming the Pantanal into a wetland. In the north, flooding occurs from March to April and from July to August in the south [2].

## 2. Experimental Section

This paper aims to provide an overview of natural disasters that occur in the Pantanal, in terms of unusual floods and droughts, based on published literature and on more than two decades of the authors’ research experience in this biome.

The hydrological station at Ladário (near the city of Corumbá) has been measuring the level of the Paraguay River in the Pantanal for more than a century, from 1900 up to 2012. Annual and pluri-annual variations in the degree of flooding can be detected. The hydrology of the Pantanal rivers is unregulated.

Data on multi-annual wet and dry seasons were obtained at the fluviometric station belonging to the Brazilian naval base at Ladário, state of Mato Grosso do Sul, (station 66825000), located on the right bank of the Paraguay River, which has operated continuously since 1900; as well as data obtained from the station located on the right bank of the Miranda River, in the city of Miranda, (station 66910000), which has operated since 1965. Data analysis and interpretation were done by means of graphics incorporating the minimum, maximum and average fluviometric rates of the Paraguay River, considering the hydrological year that starts on October 1st and ends on September 30th of the next year, and also by means of graphics that take the peaks of flooding and droughts and number of years with these occurrences. The objective is to discuss the consequences of these inter-annual events for wildlife as well as their effects on human settlements, and impacts on socio-economic activities.

**Figure 1:**
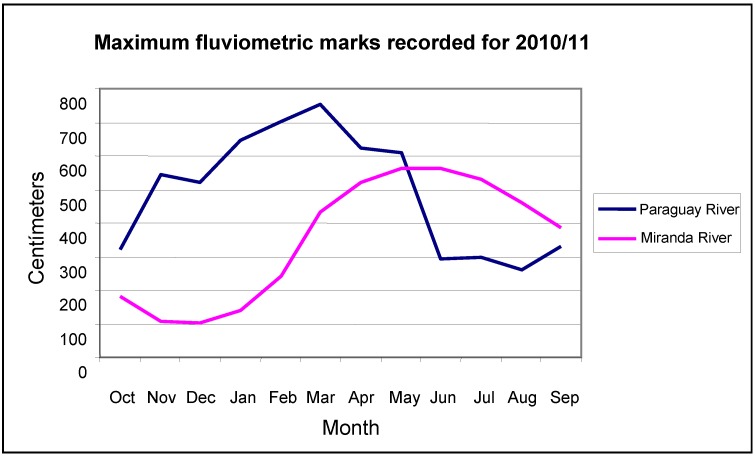
Fluviometric marks recorded on the Paraguay River at Ladário station, and on the Miranda River at Miranda city, for the 2010/2011 hydrological year of the Pantanal.

After receiving the contribution of its tributaries in Brazilian territory, the Paraguay River at Ladário has two flood cycles. The first is influenced by the rivers Aquidauana, Miranda, Negro, and Taquari, with floods in February and March. The second is influenced by the discharge of the upper Paraguay from April to June [5]. Figure 1 shows that this event may be observed for the hydrological years 2010–2011. The tributaries of the Paraguay River that flow east-west through the Pantanal floodplain are influenced directly by rainfall that starts in October. The pluviometric station located in the city of Miranda is within the limit of the transition plateau/floodplain soon after this has received the first rains that influence the overflow of the river banks, inundating the floodplain, characterizing the flood of November through June, and a period of drought from August to October. Usually, the Pantanal drought period regulated by the Paraguay River at the Ladário station goes from March to September, and may exceptionally continue to reach the month of October. The flood season runs from October through February.

## 3. Results and Discussion

Changes in the length and severity of floods and droughts potentially affect wildlife since severe floods can cover areas where vegetation may be changed in terms of community structure, population size and phenology, with consequences for both wild and domestic animals.

Despite the ample compendium from the published literature showing that flooding occurs and that the levels of floods and droughts are not regular in frequency and intensity, there have been few studies about quantified data to show how extensive floods or droughts affect the density or how this translates into population parameters of the regional wildlife. The Pantanal’s seasonal hydrological changes have their greatest impact on the region’s wildlife when the regime of floods and droughts is unusually severe.

### 3.1. Historical Records of Floods and Droughts

**Figure 2:**
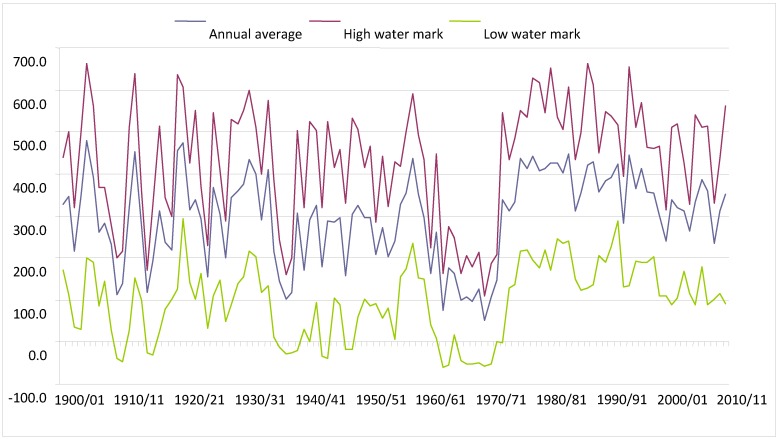
Historical events of flooding in the Pantanal during the last 111 years (January 1900 through November 2011), observed on the Paraguay River recorded by the flood gauge of the fluviometric station at Ladário.

Historical records collected at Ladário (Figure 2) show that during the period from 1962/1963 to 1972/1973 in the Pantanal was unusually dry. This was then followed by a long wet period which has lasted until the present day. During the 11-year dry period, there were no records of flooding, except for 1965–1966, when 16 cm of water was observed above the zero mark (82.4 meters in relation to sea level), and the majority of the flooding peaks were observed between one and two meters.

Before the hydrological period of 1962/1963, two hydrological periods were observed in the Pantanal: the first from 1900/1901 to 1939/1940, and the second from 1940/1941 to 1961/1962. Peaks of floods and droughts were notably variable until 1939/1940, with wide annual as well as inter-annual oscillations, with the average mark varying from one to five meters. During that period there were four records of marks above six meters, and the second greatest flood that occurred in the Pantanal was observed in 1904/1905, when the flooding reached 6.62 meters. While there were peaks of inundation, there were also nadirs of droughts with records of negative marks during the periods from 1908/1909 to 1911/1912; from 1914/1915 to 1917/1918; and from 1935/1936 to 1939/1940. 

Between 1940/1941 and 1961/1962 the Pantanal experienced an extended, 22-year, period of droughts, with an average mark at Ladário station varying from 1.6 to 4.4 meters. During this period of time, five years of drought occurred with the water level near three meters at Ladário. The variation between the minimum and maximum mark during this period is lower than those of the previous period, even when the nadirs that occurred between 1941/1942 and 1943/1944 to 1944/1945 and 1947/1948 and 1948/1949 are considered. 

Since 1973/1974 the Pantanal has been experiencing a period of inundations, with the flood gauge reaching its peak at Ladário in 1988, with a 6.64-m reading. This was considered the Pantanal’s greatest-ever inundation. This flood period, which has already lasted for 38 years, is the longest recorded for the region and is different from the previous periods in that the freatic water (the upper surface of the soil which forms the water table) has risen throughout the entire year, which results in lower intra-annual and inter-annual variation in droughts and floods. Furthermore, the position of the average river water mark has remained between three and four meters and the minimum mark between one and two meters for the greater part of the year. 

**Figure 3:**
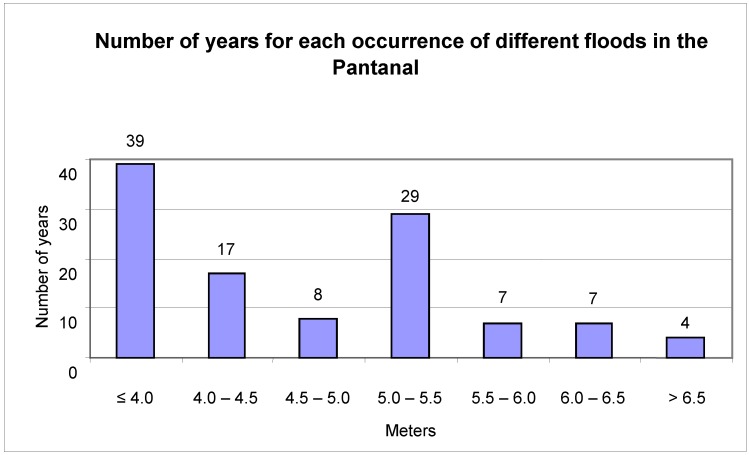
Number of years that floods occurred in the Pantanal, as recorded on the Paraguay River at Ladário Station.

**Figure 4:**
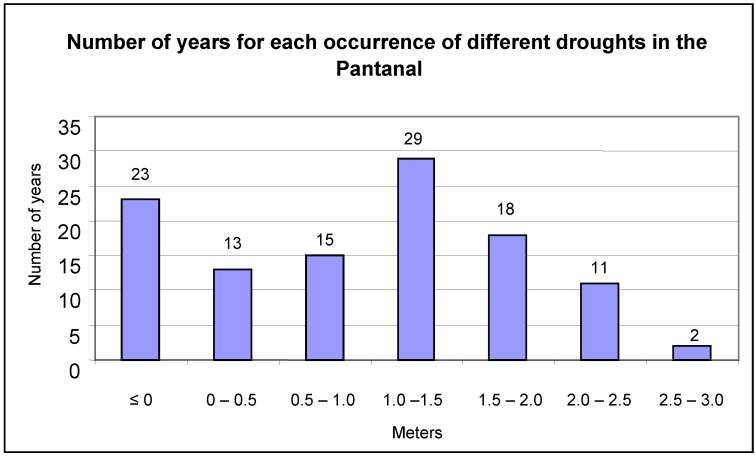
Number of years that droughts occurred in the Pantanal, as recorded on the Paraguay River at Ladário Station.

The flood mark of the Paraguay River at Ladário is four meters. This was exceeded in 72 of the studied years, or 65% of the analyzed period (Figure 3). Extreme floods, beyond 6.5 meters, were recorded for only four years (1905, 1982, 1988 and 1995). Mark zero or negatives were recorded in 23 of the years or 21% of the studied period (Figure 4). 

**Figure 5:**
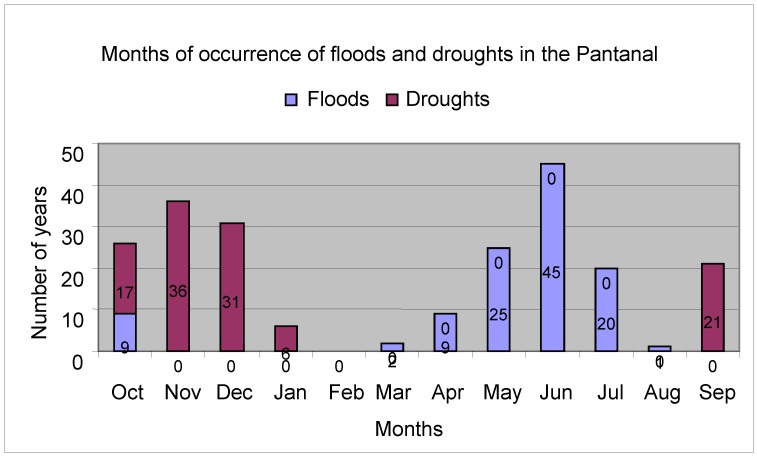
Months of occurrence of peaks of floods and droughts.

In connection with the peaks of floods and droughts, taking the data collected from the Paraguay River at Ladário station, during 111 years of observations (Figure 5), 45 flood events occurred in June (45 years), May (25), July (20), October (9), April (9), March (2) and August (1). Drought-level minimum values were observed in November (36 years), December (21), September (21), October (17) and January (6).

For an area with alternating cycles of drought and flood, such as the Pantanal, the likely consequence of this is an amplification of existing extremes and a disappearance of the current wildlife-friendly flux equilibrium.

Current studies on a global scale have shown that greenhouse gases are making water-poor regions even poorer, while increasing precipitation where it is already relatively high [6].

In connection with the phenomenon El Niño and the occurrence of floods in the Pantanal, the present information indicates a relationship between droughts and El Niño events and another between floods and La Niña occurrences [7]. This study shows that since 1900, in the 30 years that El Niño has occurred, no severe flood was observed in the Paraguay River (at the Ladário fluviometric gauge station) associated with the El Niño phenomenon. Based on this trend, it was predicted that in 1998 El Niño-associated flood would occur, and, indeed, a moderate flood occurred that year (Paraguay River maximum level reaching 4.64 meters at Ladário station).

Analysis of fluviometric data from Ladário, since 1900, revealed a flooding periodicity which fluctuated around a seven-year cycle of severe flooding and a seven-year event of moderate flooding in the period between 1900 and 1960 [8]. For fourteen years, from 1960 until 1974, the floods in the Pantanal were very moderate, but in 1974 there was a severe flood. 

Over the next 24 years, from 1974 until 1998, this 7-year flooding cycle was observed in the Pantanal. For the Paraná River basin a tight correlation has been observed between the occurrence of El Niño and the extent of river flow. For this basin of the upper Paraguay River, this is less evident, even though the catchment area of this river forms of the Paraná River basin as a whole [9]. In the southern Brazil and throughout Uruguay the relationship between El Niño and precipitation is positive. Another study points out that the occurrence of El Niño influences the droughts in the Upper Paraguay River Basin and La Niña influences floods in the Pantanal [10]. During the period of 1940–1975 each occurrence of El Niño was followed by a drop in the fluviometric marks at Ladário. It is also remarkable that the well known El Niño event of 1997 was followed by a severe drought in the Pantanal. On the other hand, each event of La Niña, between 1960–1997, was followed by wet seasons in the Pantanal [10].

### 3.2. Effects on Wildlife

As a wetland ecosystem, the Pantanal is characterized by an indistinct and ever-changing boundary between water and land. The variety of habitats present is great. Since their seasonality in production of food and other ecological resources is off-set, a great number of animals can thrive with minimal competition. The dynamic patterns of floating vegetation, annual plants, solid ground and open water provide ample niches for a wide range of plants and animals [1,2,3,4,5,6,7,8,9,10,11,12,13,14].

Every year, many parts of the Pantanal change from terrestrial into aquatic habitats and back. From May to October the land dries out and grasslands and scattered pools appear. The most striking aspect of the Pantanal is its curious combination of hydric and xeric vegetation growing side by side. Several case studies carried out within the Pantanal support the relationship between flooding patterns and nutrient and life cycles with different emphases on: (a) seasonal succession of aquatic macrophytic vegetation communities [15]; (b) arboreal communities [14,16,17]; (c) structure and dynamics of young trees [18]; (d) production of fruits by the palm *Bactris glaucescens *[19]; (e) aquatic habitats, limnology, water chemistry, primary production and morphological patterns [20,21,22,23,24,25].

The diversity of benthic macroinvertebrates (zoobenthos such as decapods, molluscs, oligochaetes, insect larvae which feed upon microoganisms and algae) was estimated at 70 taxa [26]. The zoobenthos is important in the food chain because its component organisms serve as food for fish, birds, mammals and other animal groups. Ostracoda and Nematoda are the two most abundant groups. Crustacean diversity is represented by 10 species, with the shrimp *Macrobrachium amazonicum *and the crabs *Dilocarcinus pagei, Sylviocarcinus australis, Trichodactylus borellianus *and *Valdivia camerani *recorded in surveys [27]. These decapod crustaceans play an important role in ecological processes of the Pantanal’s aquatic ecosystem since they participate in the trophic chain as herbivores, predators and decomposers, and are prey for fish and other animal groups.

Diversity of aquatic macrophytes in the Pantanal varies from the smallest (*Wolffia brasiliensis*) to the largest hydrophyte, *Victoria amazonica*. There are at least 280 species of aquatic macrophytes in the Pantanal, exhibiting various degrees of dependence on water. The most species-rich families are Poaceae (26 species), Cyperaceae (19), Fabaceae (15), Onagraceae (15) and Pontederiaceae (12), and the most species-rich genera are *Ludwigia* (15 species), *Bacopa* (12), *Utricularia* (11), *Nymphaea* (7), and *Polygonum* (7) [12]. Considering only the Pantanal plain, 1,863 species of phanerogams are known, legumes being the most numerous (240 species), followed by more than 200 grasses [28]. The phanerogam list could be updated to nearly 2,000 species [29]. Because of their adaptations to flooded areas, herbaceous forms are the most numerous (around 1,000 species). They mostly occupy seasonally flooded and grassland habitats [28].

Because of its central position in South America, the Pantanal is an encounter point for a number of phytogeographic provinces, including Amazonia (to the North), the Cerrado (to the East), the Atlantic Forests (to the South), and the Bolivian and Paraguayan Chaco (to the West). This favors a great diversity of vegetation types. There are various degrees of Cerrado cover: more than 70% in the regions of Aquidauana, Barão de Melgaço and Paiaguás (central and eastern part of Pantanal); between 40 and 50% in the regions of Cáceres, Nhecolândia and Miranda; and 10% in the region of Poconé), whereas *Cerrado* vegetation is absent in the regions of Nabileque and Paraguay—with dominance of Chaco and Amazonian vegetation, respectively; a synthesis of vegetation types is found in the literature [13].

There is a significant difference between the most humid months and the drier months of the hydrological year in wet biomass of native pastures [30]. 

Aquatic plants respond well to flood dynamics [12]. Many shallowly flooded grasslands dry up and the seasonal aquatics periodically disappear, among them even perennials, such as *Pontederia parviflora* and *Sagittaria guayanensis*, as do small annuals like *Bacopa* spp. and *Echinodorus tenellus*. In the Pantanal itself there is a stronger wet and dry cycle, whereas on the upper watershed the flood pulse is much lower, even though the area has 50–100% more rainfall. Here soils remain waterlogged or retain a high water table, all year round. This occurs because the area is fed by ground water in the dry season, flowing over an impermeable layer of laterite, making the water ferruginous, or over basalt or sandstone. Plant distribution in *cerrado* wet grassland and *vereda* is related to ground-water level [31], yet in the Pantanal the water table may fall below two meters in the dry season, hence above-ground water level in the wet season is a more important factor. In some parts of the Pantanal landscape these changes in dry phase and aquatic phase are more pronounced, as reflected in the high proportion of opportunistic therophytes [32] on intermediate ground between floodless and deeply flooded stretches. *Vereda* soils are more organic and peaty, acting like a water-storing sponge. These soils are organosoils and gleysoils [33]. The soils in the Pantanal, even though also hydromorphic vary from pure sand to heavy clay. However, due to the very flat landscape, in the dry period the water table reaches the surface only in low-lying parts, for example in ponds and water courses.

Natural pastures of the Pantanal are mainly formed by species such as *Axonopus purpusii Mesosetum loliiforme *and *Panicum laxum *covering the open fields and sandy soils of the floodplain. Other plant species forming homogeneous fields are present, such as the legume *Desmodium barbatum *and the *fura-bucho* genus *Paspalum*. Since these fields of natural pasture remain submerged during the wet season, cattle ranchers are introducing exotic grassland species on the higher ground of the Pantanal. Cultivated pastures have been expanding rapidly in the floodplain to compete with the ranchers located in the upper Cerrado plateaus surrounding the Pantanal. Two exotic species dominate such pastures: *Urochloa decumbens *(formely *Brachiaria decumbens*)and *Urochloa humidicola *(former *Brachiaria humidicola*)*. *They aggressively cover the ground and have been widely used to convert natural vegetation into cultivated pastures [34]. 

Up to 2008, the Pantanal had lost 12.1 % of its original forest cover, a comparatively low figure in the modern world [35]. However, at the current rate of forest loss, the natural vegetation of the Pantanal will have been totally destroyed by 2050, if no measures are taken to combat this trend.

Not only is the Pantanal threatened by loss of natural habitat, but also by more subtle change to existing communities. These will be explored in the following paragraphs. One potent potential impact is alterations in flooding regimen. This can, in its turn, change the structure and composition of plant communities. This can have collateral impacts on the fauna that depends on that particular kind of vegetation or microhabitat. A single example: a study on the riparian forests of the Paraguay River which studied vegetation mortality after the exceptional flood of 1995 found tree mortality rate to be 4.1%. Trees with diameter at breast height >5 cm were sampled in 108 (10 × 10 m) plots in 1994, and re-sampled in 1996 [36]. Tree mortality increased with the increase in topographic positions, at community level, suggesting that places where the flooding is less frequent are more affected by extreme floods.

Islands located in dry areas of the Pantanal (in the alluvial cone of the Taquari River), have shown alterations in the ecological communities, modifying the local landscape units [37]. These islands are subjected to floods that cover the local vegetation, depending on the intensity of flooding. According to this study, these floods have been variable in the region year after year since 1974. In addition to alterations in the natural vegetation, impacting the local flora and fauna, the inundations also impact the local economy, with deactivation and abandonment of numerous rural properties and human settlements along the river. 

The speed of such changes is illustrated by a study [38] made near the Paraguay River between 1966 and 1995. In the region the water flow of the Taquari River had become seasonally impounded. Open fields became flooded and were colonized by *cambará *tree (*Vochysia divergens).* This tree can form mono-specific stands, known locally as *cambarazais*. These have become locally common due to long-term flooding of old field areas. 

Frequent and unusual pluri-annual flooding events recorded in the Pantanal have been responsible for the sudden colonization of these trees in seasonally flooded open areas [38]. During the occurrence of longer dry and wet successive periods, two trends have been observed: (1) during longer dry events some woody plant colonize the seasonally flooded open fields, which usually are covered by herbaceous vegetation; (2) during longer wet events, flood tolerant trees, mainly *V. divergens*, form homogeneous plots expanding riparian forest. 

Changes in the cycles of flood and drought can also affect the Pantanal fauna. The abundant Pantanal crocodilian species *Caiman crocodilus yacare* exhibits seasonal variations in diet, foraging behavior, and in habitat selection during the hydrological year of the region [39]. A relationship between rainfall, nesting habitat and fecundity of the Pantanal caiman has been found [40]. Terrestrial activities of caimans, exhibiting coordinated movement during the dry season in the Pantanal, have been reported [41].

The ecology of breeding birds is intimately associated with the pulses of water availability, as illustrated below.

Most waterfowl species exhibit synchronized reproduction, where huge colonies of birds, such as wood stork (*Mycteria americana*), egrets (snowy egret *Egretta thula*, great white egret *Casmerodius albus* and the capped heron *Pilherodius pileatus*) and others, such as the spoonbill *Ajaia ajaja, *concentrate in nesting sites in the gallery forest, during the dry season, to take advantage of the seasonal resources available. The breeding colonies are formed by hundreds of nesting birds following a pattern of species breeding at the same site or in favoured trees. 

Colony-based reproduction is successful thanks to the many pairs of eyes that remain vigilant in the frequent presence of predators. The principal flying predators are: the crested caracara hawk *Caracara=Polyborus plancus*, the great black hawk *Buteogallus urubitinga *and the black vulture *Coragyps atratus. *Both female and male storks incubate and take care of the hatchlings. They feed the young about six times a day during the first three weeks. The parents catch prey in shallow waters (15–50 cm deep): mainly fish, molluscs, crustaceans, amphibians, reptiles, and insects. The colonial nesting site is active every year and the young storks born there return 3–4 years later, as adults, to reproduce in the same nesting site. 

The birds take advantage of the progressive corralling of fish and invertebrates in ponds. The aggregation of birds in colonies concentrates nutrients via the falling of feces, prey and hatchlings to the ground which, in turn, attracts predators such as caimans, anacondas, wild foxes among others. As a result, the shallow water of the dry season presents locally high turbidity, elevated levels of nitrogen and phosphate forms, as well as modified dissolved levels of oxygen, enriching the environment with nutrients.

Two waves of breeding species are known to use the same trees: a white colony and a black colony. In the sub-region of Barão de Melgaço, during the dry season, in the period of July to October, the white colony is established in a few selected trees of the Cuiabá River gallery forest, in the Porto da Fazenda, near ponds and flooded areas, with 600 nests concentrated in a single nesting site. This white colony is composed first of hatchlings of the wood ibis *Mycteria americana*, followed by lower numbers of hatchlings of the egrets *Ardea alba* and *Egretta thula*, and finally by hatchlings of the spoonbill (*Platalea = Ajaia ajaja*). The black colony is composed of the cormorant *Phalacrocorax brasilianus = olivaceus*, followed by the anhinga *Anhinga anhinga*, and the white-necked heron *Ardea cocoi.*

High densities of black-bellied tree-duck *Dendrocygna autumanalis*, white-faced tree-duck *Dendrocygna viduata,* Brazilian duck *Amazonetta brasiliensis*, and Muscovy duck *Cairina moschata *are observed. Other common birds are the southern screamer *Chauna torquata*, and macaws, including the hyacinth macaw *Anodorhynchus hyacinthinus*.

There is a clear linkage between the flooding regimen of the Pantanal and the availability of food for the adult jabirus to raise their young. The birds need low water, especially in lagoons and ponds, in order to obtain the food they can catch with their specialized beaks. The preferred food is *mussum* fish (*Symbranchus marmoratus*), which can stay dormant and encapsulated in the mud throughout the dry season, to swim again when the water rises in the rainy season. The jabiru is a specialist in detecting and catching the dormant fish in the muddy bed of the drying pond. They also catch snails (*Pomacea* spp.).

Jabirus are not present in the Pantanal during the flooding season. They migrate to higher ground to still unknown sites. Unusual rain and flooding occurred during the nesting season (dry season) of 1992. In the Nhecolândia sub-region, the hatching success was zero, from 70 nests monitored by *Projeto Tuiuiú* in July, that is, no young were seen that year. On the Taquari River, an area of 600 km along the river was surveyed and 62 nests monitored in the same year. Only one chick was born. Apparently, the incubating behavior of the parents depends upon the availability of food for the birds, which depends upon the flooding regimen. Clearly, reproductive success varies with the flooding schedule.

In 1995 (September–October), while conducting fieldwork in Cáceres sub-region, in the Ecological Reserve of Taiamã, and Porto Murtinho sub-region, a successful nesting season for jabirus was observed (Cleber Alho, personal observation). Dozens of nests were seen with three young in each nest and no abnormal flooding was reported for that season. The young jabirus are ready to leave the nests in October-November, coinciding with the period of the lowest water.

Different flooding patterns have different impacts on nesting activities. For example, the unusual flooding in July–August 1992 in the Taquari area suggests that reproductive activities in that area occurred later than those in the region of Aquidauana and Miranda. The production of young was normal in the Aquidauana/Miranda area and no chicks were born in the Taquari area, due to the abnormal local flooding in that sub-region. Studies [42,43,44] report that the stork *Mycteria americana* also exhibited nest abandonment behavior due to flooding cycles at Rio Vermelho. Field study has shown that the inter-annual variations in flooding extent can instigate dramatic changes in the number of active jabiru nests. Since the jabiru stork responds negatively to drier conditions in the Pantanal, direct human-induced changes in the hydrological patterns, as well as the effects of global climate change, may strongly jeopardize the population in the region [45]. 

Some 40 migratory bird species annually use the Pantanal. Most arrive from the northern hemisphere [46]. There are inter-American migratory routes connecting bird movements to the Pantanal. During the dry season, from May to October, migrant birds from North America fly over the Pantanal and the resident aquatic birds are more visible, concentrated in retained waters in ponds and depressions. It is evident that the seasonal change in the region is a key factor affecting migrants and resident birds and other ecological interactions [47].

As well as affecting birds, seasonal fluctuations in the amount of available food have an impact on many aspects of the behavior and ecology of many Pantanal wild mammals, including the capybara (*Hydrochoerus hydrochaeris*), the largest semi-aquatic and herbivorous rodent, very abundant in the Pantanal. Some of the foods preferred by capybara that are richer in protein tend to be more seasonal than poorer food items. There is a period of the year, from June until November, when the standing crop on lower areas susceptible to flooding is abundant and is consumed by capybaras. During the remainder of the year these food items are very scarce. The times of food abundance and scarcity are predicted by the flooding pattern [2].

Group sizes and social structure influence capybara social behavior. As soon as male sub-adults begin to reach sexual maturity, the dominant males exclude them from the social groups. The group size increases from the beginning (rainy season) to the middle of the year (dry season). During the floods the groups subdivide and are largely confined to forest patches, while in the dry season more animals are observed feeding on the pasture of the grassland. During the latter period, the groups contain young [2]. Capybara groups have larger home ranges and core areas during the dry season than during the rainy season, a change associated with a reduction in grassland area due to flooding.

Higher densities of other mammal species occur during the dry season (August and September), when there is a considerable expansion of terrestrial habitats, mainly seasonally flooded grassland, due to the shrinking-and-expansion of habitats related to the flooding pulse [48].

The marsh deer *Blastocerus dichotomus* is the largest deer in South America, with males reaching a height of up to 115 cm at the shoulder. It occurs over a wide area: northern Argentina, Paraguay, Bolivia, southeast Peru and Brazil (Amazonia, Cerrado, part of southern Brazil, and the Pantanal). It is listed as an endangered species, but it occurs at high population densities in the Pantanal [49].

All vegetation communities in which marsh deer have been observed are frequently flooded during the wet season (most habitats are formed by aquatic plants) [49]. These floristic communities occupy about 18% of the open habitats and 12.8% of the surveyed area of Nhecolândia ranch during the flood season, while the marshland, totally flooded, is completely occupied by aquatic vegetation (floating mats, *Eichhornea*, *Nymphaea, Reussia* and other plants). The vegetation type most used by marsh deer is *Andropogon* grassland, and other open areas are dominated by *Pontederia, Scleria, Nymphaea, Eleocharis, Thalia, Axonopus, Oryza, Nymphoides* and *Luziola* communities. Marsh deer select about 35 plant species, mainly aquatic plants, including *Pontederia cordata* (including both flowers and leaves), *Thalia geniculata* (mainly flowers), *Nymphaea* spp., *Aeschynomene sensitiva*, *A. fluminensis, Discolobium pulchellum, Reussia *spp. *Leersia hexandra* [49].

According to Tomás [49], the ecological density of *B. dichotomus* (the density in suitable habitats such as marshlands) is 3.12 individuals/km^2^. In other areas the estimated density is 0.73 deer/km^2^. Other estimates range from 0.09 deer/km^2^ (low flooding habitats) to 0.38 deer/km^2^ (highly inundated habitats), for dry season surveys. Seasonal flooding causes significant variation in the population distribution and density. During the dry season, deer prefer the boundary between the flooded areas and the drained range of the marshland. During the flood season, the animals are dispersed.

The black howler monkey *Alouatta caraya* inhabits the canopy of riparian forests of the Pantanal. This arboreal monkey is a generalist herbivore; it eats new shoots, leaves, flowers and fruits, altering the components and their proportions in its diet depending on the phenological peaks in the riparian forest productivity. 

Small mammals are more specialized in relation to habitat requirements, and so they are able to exploit different parts of the habitat mosaic of the Pantanal.

Local diversity and number of individuals of small mammals also depend on the available resources and behavioral specialization regarding habitat components. On the small scale, specialized species are able to exploit the spatial and temporal variation of the habitat heterogeneity, including microhabitat components [11,50]. Community differences appear to be a function of local mosaic factors. Among small mammals, like marsupials and rodents, there are habitat generalists occurring in more than three types of habitat (pan-habitat species) and habitat specialists, showing a high degree of fidelity to habitat [11]. It is common to find small mammal species with a preference for arboreal or forested habitats or for open habitats within the same study area. In the semi-deciduous forest habitats, surrounded by open savanna habitats, woodland-dwelling genera such as *Cerradomys *and *Oligoryzomys* occur within a few meters of transition zone-habitat dwellers such as *Clyomys *and *Trichomys*. The genus *Oligoryzomys *also occurs in both open and forested habitats. 

Microhabitat use among small mammals in the Pantanal shows that two genera rodents—*Oligoryzomys* (with two species occurring in the Pantanal: *O. chacoensis *and *O. microtis) *and *Cerradomys (C. subflavus)*—are more generalized in their use of microhabitats than are two echimyid species—*Clyomys laticeps* and *Trichomys pachyurus *(included in *T. apereoides *by some authors, but now recognized as a distinct species based on chromosomal differentiation [51]). *Oligoryzomys *species are broad habitat generalists of the Nhecolândia sub-region of the Pantanal. *Cerradomys subflavus* selects the savanna patch of arboreal vegetation, *capão de cerrado, *as its microhabitat. Both *Clyomys *and *Trichomys *are restricted to transition microhabitat. Although both genera co-occur in the same microhabitat, competition is avoided since *Trichomys *is scansorial while *Clyomys *is fossorial [50]. In general the transition between arboreal habitats and open areas is selected by *Trichomys pachyurus *[52]. 

Studies carried out in habitats surrounding the Pantanal, on plateaus of Chapada dos Guimarães, state of Mato Grosso, found that the combination of vegetation type and substrate structured the community of 19 terrestrial small mammal species was structured by vegetation type and substrate into several smaller communities with little faunal overlap [53]. The study showed that most species were captured in only one or two of the seven qualitative habitat types. There were open habitat species completely absent from forest (*Oligoryzomys microtis *and *Oxymmycterus roberti*), and other species that were captured only in forest habitats (*Rhipidomys macrurus, Oecomys bicolor *and *Proechimys longicaudatus)*. Cluster analysis of the 19 studied species confirmed the separation made by qualitative classification of habitats based on plant species composition and other habitat characteristics. The results for habitat associations of small mammal species determined by cluster analysis of soil and vegetation structural characteristics (independently of plant species composition) generated five fairly distinct clusters.

The gallery forest cluster grouped the same set of species that had previously been assigned to gallery forest (*Neacomys spinosus*, *Hylaeamys megacephalus*, *Nectomys squamipes*, *Oecomys bicolor*, *Proechimys longicaudatus*, and *Caluromys philander*), confirming the earlier analysis. The cluster analysis also grouped the six species that had previously been associated with wet campo (*Oligoryzomys microtis—*occurring at the Cerrado-Amazonia contact zone, *Oligoryzomys nigripes *(=*eliurus*), *Cerradomys subflavus *species group, *Necromys lasiurus*, *Monodelphis domestica*, and *Marmosa murina)*. The grouping of species was essentially the same whether it was done qualitatively by habitat type or by a quantitative analysis of structural aspects of the vegetation and substrate of the habitat [53]. 

Small mammals in the Pantanal habitats exhibit different patterns of population increase and reproductive activity, with fluctuations between the dry and wet seasons, depending upon availability of ecological resources (food, reproductive niche and space). Wild mammals respond to seasonal shrinking and expansion of habitats due to flooding regime of the Pantanal, with highest abundance of species observed during the dry season (August and September), when there is a considerable expansion of terrestrial habitats, mainly seasonally flooded grassland [48]. Animal abundance (in terms of observed individual frequencies) varied during the dry and wet seasons, and seasonally flooded grassland was the most utilized habitat by mammals in the dry season.

### 3.3. Effects on Human Settlements

Human socio-economic activities in the Pantanal, such as cattle ranching, ecotourism and fishing, may be negatively affected by severe floods and droughts. In addition, human interventions in the natural environment may contribute to these events, causing negative impacts, among these are hydroelectric plants, fire and cattle farming.

River flow is altered by hydroelectric plants. It is noteworthy that, in the Pantanal, three quarters of these enterprises involve small hydropower plants (PCHs, is the Portuguese acronym), located or planned for the upland region surrounding the Pantanal, together resulting in an important impact. Even small power-plants, which may not form a reservoir, can alter the discharge of nutrients and suspended matter and hence the cycling of nutrients in affected water bodies [54]. Furthermore, the presence of a physical barrier in the form of a dam is known to prevent the movement of migratory fish species during the spawning season, affecting fish production in the medium and long term [54]. All these changes and negative impacts on the aquatic ecology of each sub-basin forming the Pantanal should be assessed simultaneously, in terms of the integrated area of the Upper Paraguay River Basin, prior to installing such projects [54].

Wild fire is a major threat. Ranchers in the Pantanal set fire to the vegetation during the dry season as a “management” technique to “clear” the vegetation not used by cattle. The fire is initially started in the grassland but due to open areas, dry vegetation and wind, the fires often spread to savannas, woodland and forest [2,55]. Removal of natural vegetation eliminates food and shelter, especially for forest-dwelling wildlife and epiphytic plants. Deforestation also increases erosion since most elevated areas have sandy soil that is easily blown or washed away by rain. 

Cattle farming is the main economic activity in the Pantanal, with consequent impact of livestock herbivory on herbaceous and woody savanna vegetation, modifying plant community function and structure and, consequently, phytomass productivity. 

## 4. Conclusions

As mentioned previously, the Pantanal should be considered a temporary wetland since inundation occurs for only a part of the year, alternating with a dry season. A similar phenomenon is observed in Amazonia, where *várzea *and *igapó* are seasonally flooded forests, with strong seasonal cycles of inundation and its lack across the year. Like them, the Pantanal pulsates once a year, but additionally exhibits a diversity of flooding areas depending on the local geomorphological characteristics as well as local level of inundation (with some areas more susceptible to flooding) and the type of vegetation or phyto-physiognomy. Some authors subdivide the Pantanal into different sub-regions [56]. During the dry season the land is colonized by wild animals, increasing reproductive behavior and population recruitment due to the offer of newly available habitats and feeding grounds. If the dry season is longer than usual, the next flooding period may impact the natural colonization of plants and animals, as well as potential human activity such as cattle ranching.

The plant ecological community of the Pantanal exhibits seasonal productivity, with plant mortality and decomposition dependent on soil water content. Therefore, severe flood and drought events impact plant production, even more during excessively wet events, when natural habitats may remain flooded for a longer period of time. The structural and functional components of the plant ecological community may change under those severe floods and droughts, in consequence affecting herbivorous animal species.

A large percentage of Pantanal animals are of Cerrado origin, contributing to the biome’s low incidence of endemism. However, regional fauna are behaviorally well adjusted to the local fluctuation of seasonal flooding in such a way that all environmental interactions reflect animal social groups, population sizes, community structure and other ecological attributes as a function of regional characteristics. The biota’s complex interactions with the abiotic components of the ecosystem, such as seasonal flooding, reflect the life history strategy within the biome. When severe droughts or drastic floods occur, consequent impacts on these ecological attributes are expected. 

Potential changes in flooding regime in the Pantanal, due to climate change and environmental threats caused by human activities, may provoke negative impacts on regional biota. This study, based on historical records of flooding levels of the Paraguay River, implies that potential natural disasters in terms of severe droughts and drastic floods, should negatively impact regional wildlife. Management procedures dealing with conservation strategies should consider these factors for public policies.

Environmental disruption due to human interference has been registered in the Pantanal and in its surrounding plateaus. These disturbances are mainly related to conversion of natural vegetation cover into pasture and field crops that have affected natural ecosystem structure and function, potentially exposing the biome to events of natural disasters. 

Major economic activities are cattle ranching, fishing, agriculture, mining and tourism. Deforestation to convert natural habitats with pastures for cattle is increasing. The consequence is loss of biodiversity, with removal of forest that eliminates food and shelter, for forest-dwelling wildlife. The main environmental threats include: pollutants that are introduced from uncontrolled use of pesticides and herbicides, contamination with mercury from unregulated gold mining, urban liquid and solid waste, including untreated sewage, introduction of invasive exotic species, unsustainable tourism, illegal hunting, traffic of wildlife, soil degradation, lack of education and environmental consciousness, and fragility of environmental organizations to enhance legislation.

Future development projects that affect the biome’s biodiversity should be taken into action through proper environment assessment routine, based on specific terms of reference and public participation. Establishment of scoping to identify which potential impacts are relevant to assess, based on legislation, international conventions, technical and scientific knowledge and public involvement, to correct analysis to license projects or to reject them. 

In the Pantanal, flooding is a natural occurrence of the annual cycle of flow that benefits the regional wildlife with seasonal productivity. However, inter-annual severe floods and droughts, as shown in this study, can impact the biome ecosystem and, in consequence, can affect the local ecological communities, causing damages to regional wildlife and livestock. These extreme inter-annual events of floods and droughts are the result of regional climate changes, but their effects have exacerbated the risks of hydrological extremes in the Pantanal, due to human intervention and land use changes, such as deforestation, with consequences even more pronounced because of human environmental disruptions.
